# Association of Type 2 Diabetes Genetic Variants with Breast Cancer Survival among Chinese Women

**DOI:** 10.1371/journal.pone.0117419

**Published:** 2015-02-13

**Authors:** Ping-Ping Bao, Zhi-Guo Zhao, Yu-Tang Gao, Ying Zheng, Ben Zhang, Hui Cai, Wei Zheng, Xiao-Ou Shu, Wei Lu

**Affiliations:** 1 Shanghai Municipal Center for Disease Prevention & Control, 1380 Zhongshan Road West, Shanghai, China; 2 Division of Epidemiology, Department of Medicine, Vanderbilt Epidemiology Center, Institute for Medicine and Public Health, Vanderbilt-Ingram Cancer Center, Vanderbilt University Medical Center, 2525 West End Avenue, Suite 600, Nashville, Tennessee, United States of America; 3 Department of Epidemiology, Shanghai Cancer Institute, 2200/25 Xie Tu Road, Shanghai, China; MD Anderson Cancer Center, UNITED STATES

## Abstract

**Objective:**

To evaluate whether the genetic susceptibility of T2D was associated with overall survival (OS) and disease-free survival (DFS) outcomes for breast cancer (BC).

**Methods:**

Included in the study were 6346 BC patients who participated in three population-based epidemiological studies of BC and were genotyped with either GWAS or Exome-chip. We constructed a genetic risk score (GRS) for diabetes using risk variants identified from the GWAS catalog (http://genome.gov/gwastudies) that were associated with T2D risk at a minimum significance level of P ≤ 5.0E-8 among Asian population and evaluated its associations with BC outcomes with Cox proportional hazards models.

**Results:**

During a median follow-up of 8.08 years (range, 0.01–16.95 years), 1208 deaths were documented in 6346 BC patients. Overall, the diabetes GRS was not associated with OS and DFS. Analyses stratified by estrogen receptor status (ER) showed that the diabetes GRS was inversely associated with OS among women with ER- but not in women with ER+ breast cancer; the multivariable adjusted HR was 1.38 (95% CI: 1.05–1.82) when comparing the highest to the lowest GRS quartiles. The association of diabetes GRS with OS varied by diabetes status (P for interaction <0.01). In women with history of diabetes, higher diabetes GRS was significantly associated with worse OS, with HR of 2.22 (95% CI: 1.28–3.88) for the highest vs. lowest quartile, particularly among women with an ER- breast cancer, with corresponding HR being 4.59 (95% CI: 1.04–20.28). No significant association between the diabetes GRS and OS was observed across different BMI and PR groups.

**Conclusions:**

Our study suggested that genetic susceptibility of T2D was positively associated with total mortality among women with ER- breast cancer, particularly among subjects with a history of diabetes. Additional studies are warranted to verify the associations and elucidate the underlying biological mechanism.

## Introduction

Breast cancer is the most common cancer diagnosis in women in China and worldwide[1]. Survival after breast cancer diagnosis is influenced by tumor characteristics such as disease stage, tumor grades, hormonal status, and treatment. Recent epidemiological studies, including our own study, the Shanghai Breast Cancer Survival Study (SBCSS) [2], have shown that comorbidities, such as diabetes and hypertension, also influence breast cancer outcomes [3, 4, 5, 6]. It has been reported that 16% to 20% of breast cancer patients had type 2 diabetes (T2D) at time of cancer diagnosis [7, 8] and this comorbidity was inversely associated with breast cancer survival [9, 10, 11, 12, 13]. Studies also suggested that several diabetes-related conditions including insulin resistance, hyperinsulinemia, and chronic inflammation, being associated with the breast cancer outcomes [9, 10, 11, 12, 13, 14, 15]. However, few studies [11, 13] have evaluated the association of diabetes on outcomes of breast cancer by estrogen receptor status, the most important prognostic factor. In addition, studies have shown that under-diagnosis of diabetes is common, ranging from 27% in the US to 60% in China [16, 17]. Furthermore, it has been recently recognized that diabetes treatments, particularly the use of metformin, may mitigate the negative influence of diabetes on mortality, and may even reduce the risk of recurrence [18, 19], making it difficult to interpret the diabetes and breast cancer outcomes associations.

Diabetes has a strong genetic basis. To date, genome-wide association studies (GWAS) have identified multiple genetic variants for T2D [20, 21]. Given the strong relationship between T2D and breast cancer survival, we hypothesized that genetic susceptibility of T2D would also be associated with breast cancer outcomes and evaluated this hypothesis in a large scale study including 6346 breast cancer patients participated in the Shanghai Breast Cancer Study (SBCS), the SBCSS or the Shanghai Women’s Health Study (SWHS).

## Materials and Methods

### Ethics statement

The study protocols were approved by the Institutional Review Boards of participating institutes, i.e., the Vanderbilt University School of Medicine, Vanderbilt University, Nashville, Tennessee, United States; the Shanghai Cancer Institute, Shanghai, China; and Shanghai Municipal Center for Disease Prevention & Control, Shanghai, China. All participants provided written, informed consent.

### Study population

Participants for the current study were breast cancer cases from the SBCS, the SBCSS, and the SWHS [22, 23]. The details of the study design and data collection procedures have been described previously [22, 23, 24, 25]. Briefly, the SBCS is a population-based two-phase (SBCS-I and SBCS-II) case-control study that recruited 3,448 patients between August 1996 and March 1998 for SBCS-I and again between April 2002 and February 2005 for SBCS-II [24], of which 90.6% provided a blood or exfoliated buccal cell sample. The SBCSS is a population-based prospective cohort study and recruited 5042 breast cancer patients between March 2002 and April 2006, of which 98% provided an exfoliated buccal cell sample [25]. The SWHS is a population-based prospective cohort study initiated in March 1997 and completed in May 2000, which focus on the cancer and other chronic disease [26]. All participants were followed for survival status and recurrence through a combination of in-person surveys and record linkages with the Shanghai Vital Statistics Registry. Because of the overlapping recruitment time, 1469 women participated in both the SBCS and SBCSS. After taking these overlaps into consideration and excluding patients without follow-up data, a total of 6346 participants remained in the present study.

Cancer diagnoses, cancer characteristics and treatment information were obtained by medical records review. All anthropometric measurements, diabetes history, and other information were collected by in-person interviews using validated questionnaires. All study interviewers were retired, trained medical professionals.

### Genotyping and candidate SNP selection

Genotyping methods and quality control procedures have been previously described [23, 24]. Briefly, DNA was analyzed by either of two genotyping platforms. Two thousands nine hundred and one breast cancer cases were genotyped using the Affymetrix Genome-wide Human SNP Array 6.0 (Abbr. GWAS). A custom-designed Illumina Human Exome-12v1-A BeaChip (Abbr. Exome-chip) was used to genotype the other 3445 breast cancer cases. All GWAS identified variants as reported in the US National Human Genome Resource Institute (NHGRI) list by August 16, 2011, were included in this chip. Samples and variants that failed the quality control were excluded.

Genetic variants associated with T2D were searched in the GWAS catalog [27] (having disease trait “Type 2 diabetes” or “Type 2 diabetes and other traits” and p value <5×10^-8^) in Asian-ancestry population, and a total of 44 SNPs were identified. Data on 39 SNPs representing 32 independent loci (linkage disequilibrium, LD r^2^<0.3) were available for GWAS data set; One SNP from each of these 32 independent loci was selected to construct the genetic risk score (GRS) as described below. For subjects who were genotyped by the Exome-chip, 16 diabetes related SNPs were directly genotyped. For another 6 diabetes related SNPs, we were able to identify proxy SNPs (with a minimal r^2^ of 0.8 with the index SNP) and then used the proxy-index haplotype (use hapmap r23 data for JPT and CHB) to determine the corresponding risk allele for the proxy SNPs. Therefore, among subjects with Exome-chip data, genetic risk score (GRS) was constructed using the 22 SNPs (16 index and 6 proxy SNPs); of which, 20 SNPs overlapped with the 32 SNPs included in estimation of GRS in the subjects with GWAS data.

### Statistical analysis

To measure the cumulative effect of the multiple diabetes genetic variants, a weighted GRS was calculated using the odds ratio of the published T2D loci to weight the T2D-related risk alleles at each locus and then summing across all T2D loci. The weighted GRS was created as $\sum_{i}^{32}\text{SNP}i\times W_{i}$ or $\sum_{i}^{22}\text{SNP}i\times W_{i}$, where SNP_*i*_ is the number of the risk alleles one subject carries in a SNP in data from GWAS or Exome-chip, respectively, and the *W_i_* is the odds ratio with T2D published previously. The weighted GRSs were categorized into quartiles separately for subjects from GWAS data or Exome-chip data based on its own distribution.

Outcomes of the study were total mortality (for the overall survival analysis) and breast cancer recurrence or breast cancer-related death (for the disease-free survival analysis). Cox proportional hazards regression models were applied to derive hazard ratios (HRs) and 95% confidence intervals (95% CI) with adjustments of age at diagnosis (treated as continuous using a restricted cubic spline with 5 knots), and disease stage. Stratified analyses by ER status (ER+, ER-), PR status (PR+, PR-), BMI (2 levels: <25, >=25), and diabetes (yes, no, unknown) were conducted to evaluate the consistency of the effects. The homogeneity of the associations across the strata was evaluated using likelihood ratio test. The associations between the GRS quartiles and breast cancer outcomes were evaluated separately by each genotype platform (Table B and Table C in S1 File), and then combined using the inverse-variance meta-analysis with fixed-effect or random-effect models as appropriate. Genotype platform specific results and combined results were reported. A sensitivity analysis by excluding the participants with extreme follow-up time (shortest 1% and longest 1%) was also performed and reported (Table D in S1 File). The association of each SNP with breast cancer survival was also investigated using similar approaches described above. The results for individual SNP and breast cancer survival analysis were shown in Table A in S1 File.

All statistical tests were based on two-sided probabilities with significance determined by *P*-value <0.05. Statistical analyses were carried out using R (http://www.R-project.org/).

## Results

After a median follow-up of 8.08 years (range, 0.01–16.95 years), 1208 total all-cause deaths and 1160 breast cancer recurrences or deaths were documented. Subjects from GWAS data and Exome-chip data had similar 5-year overall survival rates, 88.23% and 88.13% respectively. Older age at diagnosis, advanced disease stage, negative ER or PR status, high BMI, not receiving radical mastectomy, receiving radiotherapy, and not receiving tamoxifen treatment were associated with worse overall survival and disease free survival (Table 1).

**Table 1:** Participant demographic and clinical predictors for breast cancer survival.

			**Overall survival**				**Disease free survival**		
**Characteristics**	**No. of Participants**		**Death, No.**	**5-year Survival, %^a^**	***P* value**			**5-year Survival, %^a^**	***P* value**
Genotype method									
GWAS	2901		605	88.23	0.35			85.12	<0.01
Exome-chip	3445		603	88.13				80.99	
Age at diagnosis (years)									
<40	409		100	86.06	<0.01			79.34	<0.01
40–49	2548		388	89.86				85.39	
50–59	1867		355	87.79				82.07	
≥60	1522		365	86.40				82.48	
Education									
None	2040		430	87.14	<0.01			83.51	<0.01
Elementary school	1764		283	89.66				86.10	
Middle/high school	2230		438	88.18				80.94	
College and above	312		57	86.86				79.04	
BMI									
<25	4111		720	89.39	<0.01			84.84	<0.01
≥25	2232		487	85.98				80.63	
Diabetes^b^									
No	5182		815	91.38	0.03			86.38	<0.01
Yes	622		117	92.37				84.82	
Unknown	542		276	52.90				54.10	
TNM stage (%)									
0-I	2111		192	95.32	<0.01			93.85	<0.01
II	3088		591	88.66				82.74	
III-iv	633		305	64.89				53.24	
Unknown	514		120	84.73				81.04	
ER status									
Negative	2035		411	86.00	<0.01			81.21	<0.01
Positive	3745		626	90.73				86.00	
Unknown	566		171	79.61				72.60	
PR status									
Negative	2334		463	86.59	<0.01			81.74	<0.01
Positive	3431		569	90.73				85.92	
Unknown	581		176	79.60				73.64	
ER/PR status									
ER-/PR-	1581		324	85.46	<0.01			81.13	<0.01
ER+/PR+	2978		484	91.10				86.58	
ER+/PR- or ER-/PR+	1195		222	88.77			82.59	
Unknown	592		178	79.65				73.65	
Tamoxifen use									
No	2387		484	86.58	<0.01			83.18	0.01
Yes	3009		506	91.42				86.44	
Unknown	950		218	81.87				65.03	
Radical mastectomy									
No	30		23	33.33	<0.01			9.52	<0.01
Yes	6276		1171	88.57				83.69	
Unknown	40		14	70.00				73.70	
Chemotherapy									
No	540		106	87.87	0.45			87.45	<0.01
Yes	5761		1086	88.35				83.07	
Unknown	45		16	71.11				72.47	
Radiotherapy									
No	4212		650	91.03	<0.01			87.82	
Yes	1943		510	82.43				74.60	
Unknown	191		48	84.29				81.42	<0.01

^a^ The survival rate was calculated by using the life table analysis method.

^b^ Women were specifically asked if they had this condition; Included the cases of diabetes by self-reported at baseline and follow-up surveys.

The diabetes GRS was not associated with ER/PR status, TNM stage, age at diagnosis but was associated with diabetes history at time of cancer diagnosis (*P* <0.01) (Table 2). As shown in Table 3, overall, the diabetes GRS was not associated with OS and DFS. Analyses stratified by ER status showed that the diabetes GRS was inversely associated with OS among women with ER- with a multivariable adjusted HR of 1.38 (95% CI: 1.05–1.82) when comparing the highest to the lowest GRS quartiles. As a contrast, in the ER+ subgroup, patients with higher diabetes GRS tended to have better survival, though this association was not statistically significant (*P* for trend = 0.45, *P* for interaction = 0.28). Additional adjustment for BMI did not change the results appreciably. No statistically significant association was observed between the diabetes GRS and DFS, regardless of ER status.

**Table 2:** Associations of diabetes Genetic Risk Score (GRS) with clinical characteristics.

	**GRS**				
	**Q1(N = 1588)**	**Q2(N = 1587)**	**Q3(N = 1589)**	**Q4(N = 1582)**	***P* value**
ER					0.86
Negative	31.99	31.32	32.79	32.17	
Positive	58.31	60.11	58.59	59.04	
Unknown	9.70	8.70	8.62	8.79	
PR					0.39
Negative	36.27	36.48	38.89	35.46	
Positive	53.78	54.95	52.24	55.31	
Unknown	9.95	8.57	8.87	9.23	
TNM					0.74
0-I	33.69	33.96	33.86	31.54	
II	49.43	48.46	47.45	49.31	
III-IV	9.26	9.51	10.26	10.87	
Unknown	7.62	8.07	8.43	8.28	
Diabetes					<0.01
No	85.64	83.11	81.81	76.04	
Yes	6.74	7.94	9.88	14.66	
Unknown	7.62	8.95	8.31	9.29	
Age at diagnosis					
	53.04(9.87)	52.61(9.95)	52.76(9.82)	52.61(9.97)	0.60

^a^ Percentage for categorical variables (ER/PR/TNM stage), mean (Standard deviation) for the variable of age at diagnosis.

^b^ Pearson’s χ^2^ test for categorical variables, Kruskal-Wallis test for continuous variable.

**Table 3:** Association of the diabetes Genetic Risk Score (GRS) with breast cancer outcomes.

**Quartile of GRS**	**Total Mortality**			**Recurrence/Breast Cancer-Specific Mortality**		
	**No. participants**	**No. of Event**	**HR (95% CI)**	**No. participants**	**No. of Event**	**HR (95% CI)**
**All Participants**						
Q1	1588	311	1 (reference)	1438	291	1 (reference)
Q2	1587	291	0.94 (0.80,1.10)	1439	276	0.94 (0.80,1.11)
Q3	1589	295	0.97 (0.83,1.14)	1431	271	0.94 (0.80,1.11)
Q4	1582	311	0.99 (0.84,1.16)	1447	278	0.92 (0.78,1.08)
P for trend			0.99			0.33
**By ER status**						
**Women with ER- breast cancer**						
Q1	508	90	1 (reference)	472	93	1 (reference)
Q2	497	102	1.33 (1.00,1.77)	454	102	1.29 (0.97,1.72)
Q3	521	96	1.05 (0.78,1.40)	476	90	0.88 (0.66,1.19)
Q4	509	123	1.38 (1.05,1.82)	476	109	1.13 (0.86,1.50)
P for trend			0.08			0.90
**Women with ER+ breast cancer**						
Q1	926	175	1 (reference)	840	156	1 (reference)
Q2	954	144	0.76 (0.61,0.95)	870	137	0.82 (0.65,1.04)
Q3	931	155	0.92 (0.74,1.15)	849	143	0.96 (0.76,1.21)
Q4	934	152	0.86 (0.69,1.07)	858	142	0.88 (0.70,1.11)
P for trend			0.45			0.53
**By history of diabetes**						
**Breast cancer patients with diabetes**						
Q1	107	17	1 (reference)	97	16	1 (reference)
Q2	126	15	0.87 (0.42,1.80)	120	15	0.77 (0.33,1.80)
Q3	157	25	1.26 (0.67,2.37)	144	27	1.37 (0.73,2.59)
Q4	232	60	2.22 (1.28,3.88)	210	47	1.51 (0.82,2.75)
P for trend			<0.01			0.03
**Breast cancer patients without diabetes**						
Q1	1360	230	1 (reference)	1225	219	1 (reference)
Q2	1319	203	0.90 (0.74,1.08)	1188	191	0.87 (0.72,1.06)
Q3	1300	205	0.95 (0.79,1.15)	1161	191	0.92 (0.75,1.11)
Q4	1203	177	0.83 (0.68,1.01)	1096	168	0.81 (0.66,0.99)
P for trend			0.12			0.07

NOTE: All models adjusted for age at diagnosis (treated as continuous, using a restricted cubic spline with 5 knots) and TNM-stage. P values for interaction between ER status and weighted GRS were as follows: *P* = 0.28 for total mortality and *P* = 0.71 for recurrence or breast cancer-specific mortality; *P* values for interaction between diabetes status and weighted GRS were as follows: *P*<0.01 for total mortality and *P*<0.01 for recurrence or breast cancer-specific mortality.

The associations of the diabetes GRS with OS and DFS also varied by diabetes status (*P* for interaction <0.01, and <0.01 for OS and DFS respectively). In women with self-reported history of diabetes, higher GRS was significantly associated with worse OS and DFS (*P* for trend < 0.01 and = 0.03 respectively). For OS, the HR was 2.22 (95% CI: 1.28–3.88) when comparing the highest to the lowest quartiles (Table 3). For the DFS, however, none of the 3 upper quartiles reached the statistical significant level, though the linear trend was statistically significant (*P* = 0.03).

Additional analyses further stratified by ER status and diabetes status were conducted and showed that the diabetes GRS was associated with increased overall survival for ER+ breast cancer patients without diabetes history (*P* for trend = 0.02) but a decreased OS for those had a history of diabetes (*P* for trend = 0.01, *P* for interaction<0.01). The diabetes GRS was associated with decreased OS among patients with ER- breast cancer, particularly those with a history of diabetes, with a HR of 4.59 (95% CI: 1.04–20.28) (Table 4). A sensitivity analysis by excluding 129 participants who had extreme short or extreme long follow up times did not change the results appreciably (Supplementary Table 4).

**Table 4:** Association of the diabetes Genetic Risk Score (GRS) with OS and DFS by ER status and diabetes status.

				**GRS**						
		**No. of Participants**	**Events**	**Q1**	**Q2**	**Q3**	**Q4**	***P* value**		***P* interaction**
**Total Mortality**										
**ER+**										
Diabetes	No	3047	429	Reference	0.69 (0.53,0.89)	0.92 (0.71,1.18)	0.65 (0.49,0.86)	0.02		
	Yes	411	70	Reference	0.49 (0.20,1.24)	0.77 (0.35,1.70)	1.75 (0.91,3.35)	0.01		< 0.01
**ER-**										
Diabetes	No	1652	261	Reference	1.54 (1.09,2.18)	1.14 (0.79,1.65)	1.34 (0.93,1.92)	0.29		
	Yes	184	38	Reference	2.52 (0.42,15.03)	3.30 (0.69,15.73)	4.59 (1.04,20.28)	0.03		0.07
**Recurrence/Breast Cancer-Specific Mortality**										
**ER+**										
Diabetes	No	3047	409	Reference	0.72 (0.55,0.95)	0.93 (0.72,1.21)	0.72 (0.54,0.95)	0.09		
	Yes	411	61	Reference	0.86 (0.26,2.92)	0.97 (0.43,2.20)	1.43 (0.69,2.98)	0.06		0.02
**ER-**										
Diabetes	No	1652	257	Reference	1.44 (1.02,2.03)	0.97 (0.67,1.39)	1.12 (0.78,1.61)	0.98		
	Yes	184	36	Reference	2.43 (0.45,13.29)	1.67 (0.41,6.86)	1.97 (0.51,7.54)	0.57		0.43

NOTE: All models adjusted for age at diagnosis (treated as continuous, used a restricted cubic spline with 5 knots) and TNM-stage.

Analyses stratified by BMI and PR status did not find any heterogeneity in the diabetes GRS-OS or diabetes GRS-DFS associations (data not shown).

## Discussion

To our best knowledge, no study, to date, has evaluated the effects of the common inherited genetic variants related to T2D on breast cancer survival. The reasonably high heritability of diabetes, the availability of GWAS-identified risk variants for diabetes among Asian [20], and the strong relationship between diabetes and breast cancer survival [9, 10, 11, 12, 13] motivated us to evaluate the association of T2D genetic variants with breast cancer prognosis. We applied the GRS to measure the combined genetic effects of T2D-associated SNPs on breast cancer survival. We found no overall association between T2D GRS and breast cancer survival among all breast cancer cases. However, among ER- breast cancer patients, higher GRS was associated with worse overall survival. In addition, we found that the association of genetic susceptibility to T2D with survival among breast cancer patients was modified by the history of diabetes. In women with a history of diabetes, higher diabetes GRS was significantly associated with worse OS. The association did not change after adjusting for other prognostic factors, including age at diagnosis, TNM stage, and BMI, indicating it was independently associated with overall survival among patients with ER- breast cancer.

While the impact of diabetes on breast cancer survival has been previously reported, its mechanism remains unclear. It has been suggested that diabetes may affect breast cancer prognosis, possibly due to increased diabetes-related comorbidity, or direct effects of insulin resistance and/or hyperinsulinemia [9, 10, 11, 12, 13, 14, 15]. Insulin is involved in the promotional stage of breast tumorigenesis and progression to metastatic phenotype[28]. In vitro studies suggested that insulin promoted growth of the ER+ human breast cancer cell lines and the effects of insulin on ER- human breast cancer cell lines are quite complex and inconsistent with limited number of cell lines[28]. The epidemiological results are not consistent for the modifying influence of ER status on the association of diabetes and breast cancer survival. Two studies, including a study conducting among early stage Asian breast cancer patients, showed that the prognostic influence of T2D on breast cancer outcome is modified by ER status [11, 13]. A large cohort study recruited 6342 early-stage breast cancer patients in the US, however, suggested that the diabetes was inversely associated with the prognosis of ER+, but not ER-, breast cancer patients[29]. Chlebowski et al. [30, 31] had suggested that insulin or IGF may be important in the ER- breast cancer subset, and the evidence has limited and more studies are needed to eliminate the influence of insulin on ER- breast cancer. A significant association of diabetes with a higher distant metastasis rate during the follow-up period of 5 years was only related to the ER- subgroup [10].

The positive association between higher diabetes GRS and the worse overall survival in women with ER- breast cancer in this study suggested that a genetic background favorable to development of T2D was positively associated with total mortality. Most of T2D genes identified by GWAS are likely responsible for pancreatic β-cell function, which likely mediate their influence on T2D susceptibility [20]. Insulin resistance is also widely accepted to be a major component of T2D susceptibility [32]. Compared with Europeans, East Asians may be more susceptible to insulin resistance [33]. Whether the increased mortality risk among breast cancer survivors with diabetes is driven by an increase in cancer recurrence or a result of competing diabetes-related comorbidities such as cardiovascular disease remains unclear. Previous studies [9, 10, 12] reported that T2D was not significantly associated with DFS, suggesting that the worse survival following BC may due to increased deaths attributed to diabetes-related comorbidities rather than effect of diabetes itself. Finding of a null association for GRS and DFS in the present study also support a role of the diabetes-related access of mortality and morbidity. The finding of an inverse association between GRS and total mortality among ER+ breast cancer patients who had no history of diabetes was unexpected and could possibly be due to chance. It also might be the influence of its effective treatment, especially for hormonal therapy, among ER+ breast cancer patients who had no history of diabetes, which weakened the association of T2D GRS and the risk of mortality. The true association can be estimated only in the ER- breast cancer,

Diabetes is treated with a variety of medications. These has been recently recognized that diabetes treatments, particularly the use of metformin, may mitigate negative influence of diabetes on mortality, and may even reduce the risk of recurrence [18, 19], making it difficult to interpret the diabetes and breast cancer outcomes associations. Unfortunately, this study did not collect information on diabetes medication before or after breast cancer diagnosis. Our findings could at least partly indicate that genetic markers of T2D may affect the ER- breast cancer outcome through some mechanisms related to diabetes alone or others, suggesting a shared genetic component between T2D and ER- breast cancer survival. Furthermore, our findings showed an interactive effect between T2D-related SNPs and diabetes on breast cancer survival. The precise mechanism for our findings is still uncertain and further studies will be needed to further explore this relationship.

Strengths of this study include the large sample size, genetically homogeneous population, prospective investigation of disease outcome, and the availability of detailed clinical characteristics of disease. However, some limitations also warrant mention. One limitation of our study is that it relies on the GWAS catalog for identification of SNPs for inclusion. The threshold for entry of SNPs into the GWAS catalog is higher than that used for most association studies, so some potentially interesting loci may have been missed. Another limitation is that not all T2D-related SNPs among Asian were captured across the entire genome and the percentage of T2D variation captured by the GWAS was low. Furthermore, not all GWAS diabetes SNPs have been included in our study; Two SNPs located on chromosome 23 were not available in our GWAS platform and no proxy SNPs were successfully identified for them, thus our GRS included limited information for these two SNPs. Similarly, in Exome-chip platform, our GRS only captured information from 22 loci. Missing information may compromise the statistical power of our study. Investigations on the molecular pathway specific GRS may provide additional valuable insights into the etiology of these two diseases. However, the hypotheses that lead to these investigations are beyond the scope of the current study. Among the 33 investigated SNPs in the current study, 15 had unclear functionality, 4 had function in insulin action and the rest 14 were related to β-cell function. Future studies with high-density genotyping on certain regions that related to these pathways may provide more convincing information.

In summary, we found that higher diabetes GRS was related with low overall survival among ER- breast cancer patients, particularly those with a history of T2D but was not significantly associated with disease free survival or outcomes of ER+ breast cancer. Further studies are warranted to replicate our findings and to investigate the underlying mechanism(s) of these diseases.

## Supporting Information

S1 File This file contains Tables A-D. Table A, Associations between 39 T2D-related SNPs in GWAS data and 22 SNPs in Exome-chip data with breast cancer outcomes. Table B, Association of the diabetes Genetic Risk Score (GRS) with breast cancer outcomes in Exome-chip data. Table C, Association of the diabetes Genetic Risk Score (GRS) with breast cancer outcomes in GWAS data. Table D, Association of the diabetes Genetic Risk Score (GRS) with breast cancer outcomes from a sensitivity analysis in which 129 patients with extreme follow-up time (shortest 1% and longest 1%) were excluded.
(RAR)
